# TGFβ1 as a Predictive Biomarker for Collateral Formation Within Ischemic Moyamoya Disease

**DOI:** 10.3389/fneur.2022.899470

**Published:** 2022-07-07

**Authors:** Yuanbing Chen, Miao Tang, Hui Li, Hongwei Liu, Junyu Wang, Jun Huang

**Affiliations:** ^1^Department of Neurosurgery, Xiangya Hospital, Central South University, Changsha, China; ^2^National Clinical Research Center for Geriatric Disorders, Xiangya Hospital, Central South University, Changsha, China

**Keywords:** moyamoya, TGFβ1, collateral, VEGF, biomarker

## Abstract

**Objective:**

Moyamoya disease (MMD) is a unique cerebrovascular occlusive disease characterized by progressive steno-occlusion within the terminal segment of the internal carotid artery. However, good collaterals from an external carotid artery are essential to compensate for the ischemia in moyamoya disease. This study aimed to investigate the transforming growth factor-beta 1 (TGFβ1) in plasma as a potential biomarker for predicting collateral formation in ischemic MMD.

**Methods:**

The transcriptome profile downloaded from Gene Expression Omnibus (GEO) was used to analyze the differential expression of genes between the ischemic MMD and the control groups. We prospectively recruited 23 consecutive patients with ischemic MMD that was diagnosed *via* digital subtraction angiography (DSA). Nine patients with intracranial aneurysms and four healthy people served as controls. The collaterals from the external carotid artery were examined using DSA. We evaluated whether the collateral formation was associated with TGFβ1 in patients with ischemic MMD. Western blot, RT-qPCR, ELISA, and tube formation assay were used to explore the relationship between TGFβ1 and angiogenesis, as well as the potential mechanisms.

**Results:**

The mRNA levels of TGFβ1 were upregulated in the patients with ischemic MMD. The plasma TGFβ1 levels were higher in the patients with ischemic MMD than in the aneurysm and healthy patients (*p* < 0.05). The collateral formation group has higher levels of serum TGFβ1 than the non-collateral formation group (*p* < 0.05). The levels of vascular endothelial growth factor (VEGF) are positively correlated with TGFβ1 levels in the plasma (*R*^2^ = 0.6115; *p* < 0.0001). TGFβ1 regulates VEGF expression *via* the activation of the TGFβ pathway within HUVEC cells, as well as TGFβ1 stimulating HUVEC cells to secrete VEGF into the cell culture media. An *in vitro* assay revealed that TGFβ1 promotes angiogenesis within the endothelial cells.

**Conclusion:**

Our findings suggest that TGFβ1 plays a vital role in promoting collateral formation by upregulating VEGF expression in ischemic MMD.

## Introduction

Moyamoya disease (MMD) is a rare cerebrovascular occlusive disease characterized by progressive steno-occlusions in the terminal segment of the internal carotid artery (ICA), as well as their proximal branches. Furthermore, the appearance of abnormally dilated compensatory collateral vasculature is revealed upon angiography (1, 2). With the progress of intracranial vascular stenosis, both the intracranial and extracranial vessels are stimulated to develop collateral circulation (3, 4). Those new collaterals are found and considered necessary to maintain perfusion in MMD (5–7). When the collateral circulation is insufficiently compensated, it induces ischemic symptoms (4).

Bypass surgery is recommended as a first-line treatment of MMD, including direct bypass, indirect bypass, and combined strategies (8–10). Moreover, direct bypass surgery can immediately increase blood flow, but indirect bypass requires more time to produce angiogenesis from the muscle and dura (8, 9). Also, there is a lack of effective collateral angiogenesis needed to prevent strokes after indirect bypass surgery immediately (11). Notably, previous research has revealed that transdural collaterals are associated with the capacity to develop collaterals postoperatively (7, 12). Therefore, a comprehensive understanding of the relevant mechanism of preoperative collateral development is expected to guide the establishment of good postoperative collateral. However, the molecular mechanisms regulating angiogenesis in the progression of collateral remain unclear and lack a reliable biomarker to predict the collateral (8, 13–15).

Transforming growth factor-beta 1 (TGFβ1) is secreted as a latent form and functions as a multifunctional polypeptide growth factor acting as a significant modulator of cellular growth and differentiation, also playing a vital role in regulating the expression of potent angiogenic factors (16, 17). There is a high level of TGFβ1 in the serum of patients with MMD that has been shown in previous research (17–19). However, it is only speculated that the expression of TGFβ1 is related to the pathophysiology of moyamoya disease, and the relationship between TGFβ1 and the collateral is still undetermined.

In this research, we sought to explore the genes specifically expressed in the intracranial arteries of MMD and identify the angiogenesis-related genes. In addition, the relationship between TGFβ1 and the collaterals originating from the dura was identified in ischemic MMD. Finally, we investigated the mechanisms of TGFβ1 underlying the regulation and development of collaterals.

## Materials and Methods

### Patients

From September 2019 and November 2021, 23 Asiatic patients diagnosed with ischemic MMD in the department of neurosurgery of Xiangya hospital were recruited for this study. MMD was diagnosed according to the guidelines for diagnosis and treatment of MMD by the Research Committee on Spontaneous Occlusion of the Circle of Willis (20). All patients underwent DSA to evaluate the transdural collaterals from the dura and agreed to have their plasma examined for potential biomarkers that were included in this study. The patients diagnosed with quasi-moyamoya disease (moyamoya syndrome secondary to identified etiologies, including a history of vasculitis, neurofibromatosis, tuberous sclerosis, hypertension, diabetes, and others) were excluded from this study. MMD was categorized into pediatric and adult groups based on age. Nine patients with intracranial aneurysms and four healthy persons were included in the study as controls. Clinical information was collected. The Research Ethics Committee approved this study of the Xiangya hospital. All study participants were given written and informed consent.

### Transdural Collaterals

**Figure 1:**
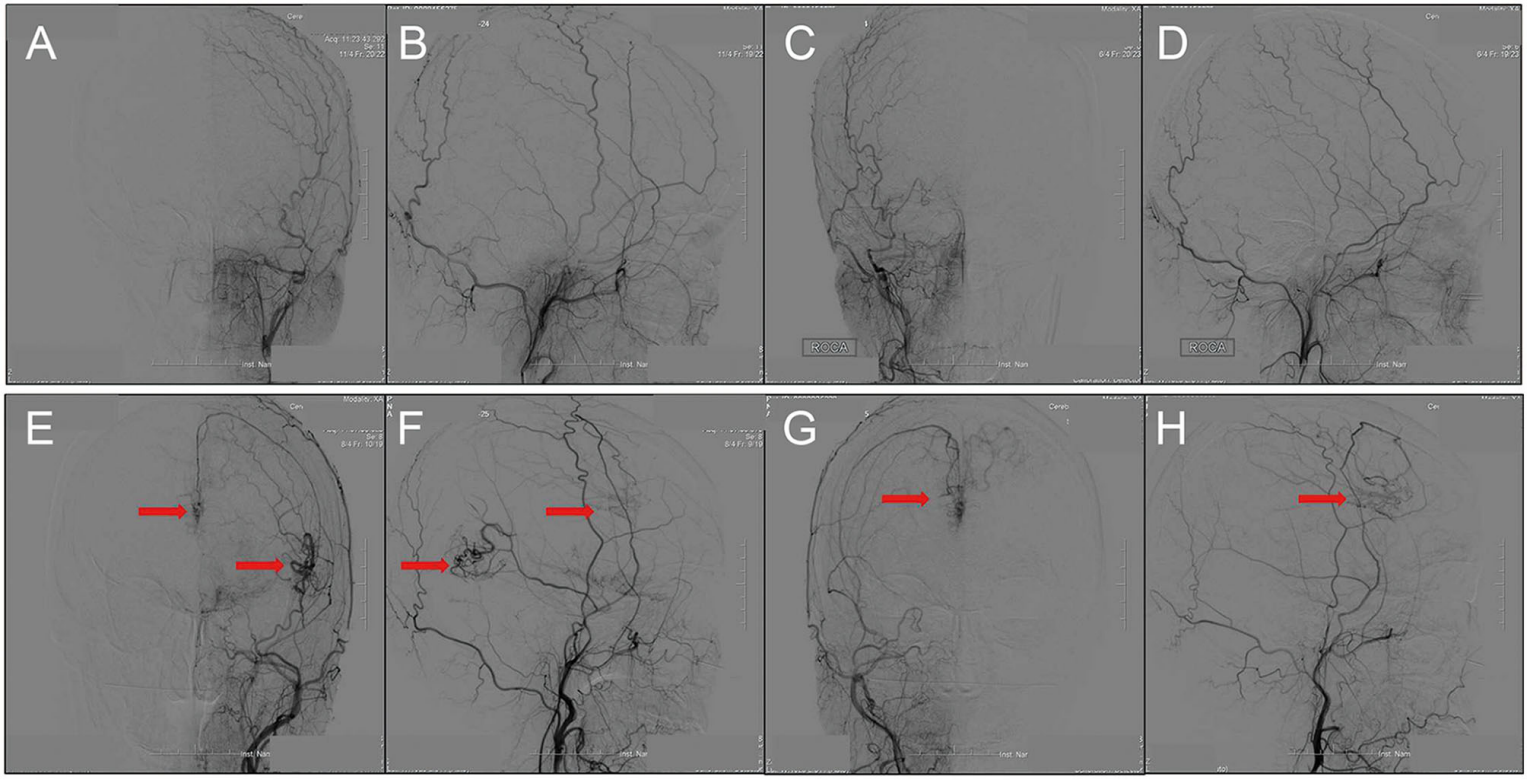
DSA showed the transdural collaterals arising from the middle meningeal artery. **(A–D)**, anteroposterior **(A)** and lateral **(B)** views of left ECA injection, anteroposterior **(C)** and lateral **(D)** views of right ECA injection. There is no MMA supplying blood to the brain cortex through transdural collateral vessels. **(E–H)**, Anteroposterior **(E)** and lateral **(F)** views of left ECA injection, anteroposterior **(G)** and lateral **(H)** views of right ECA injection from the other patient. The MMA provides transdural collateral vessels to the brain cortex (red arrowheads).

Transdural collaterals are defined as the blood supply to the cerebral cortex from the external cervical artery (ECA). To detect the blood supply from the ECA more accurately, we only selected the middle meningeal artery for evaluation. Transdural collaterals are considered if obvious blood supply from the middle meningeal artery (MMA), according to the DSA (including bilateral ECA injections), is seen (Figure 1).

### Bioinformatics Analysis

For differentially expressed angiogenesis-related gene screening, the Gene Expression Omnibus (GEO) dataset (GSE157628) was obtained from the GEO database (http://www.ncbi.nlm.nih.gov/geo). The MCA specimen was collected during the STA–MCA anastomosis (21). Data analysis was carried out in the R environment using the limma package. Both upregulated and downregulated differentially expressed genes (DEGs) were used for further study, and statistical significance was set at *p* < 0.05, |log2 fold change|≥2 in this study. The DEGs were performed for gene ontology (GO) functional enrichment analysis.

### ROC Curve Analysis

To evaluate the predictive value of the biomarkers (TGFβ1 and VEGF), the receiver operating characteristic (ROC) curves were conducted to calculate the AUCs. The sensitivity and specificity of the biomarkers' predictability in their capacity to develop transdural collaterals were assessed by calculating the AUC value of the ROC curve using SPSS 21.0 software.

### Enzyme-Linked Immunosorbent Assay

Patient plasma was collected before surgery and stored at −80°C. The experimental procedures were carried out according to the manufacturer's instructions for the TGFβ1 (4A Biotech Co, #CHE0029, China) and VEGF (4A Biotech Co, #CHE0043, China) ELISA kit.

### Cell Culture

Human umbilical vein endothelial cells (HUVECs) were purchased from the American Type Culture Collection (ATCC). HUVECs were cultured in HUVEC complete medium (CellCook, cat: CM2007, China) supplemented with 10% fetal bovine serum (FBS), 100 U/ml penicillin, and 100 U/ml streptomycin, and maintained at 37°C in a 5% CO_2_ atmosphere. HUVECs were collected and seeded at a density of 1 × 10^6^ cells/well into 6-well-plates. After 4 h, the non-adherent cells were removed and added to the HUVEC complete media (2.5 ml). The TGF-β1 (Cusabio, China) was added to the media with a final concentration of 5 ng/ml. After 24 h, the medium and cells were harvested.

### Western Blot

Human umbilical vein endothelial cells were precipitated by centrifugation and lysed in immunoprecipitation (IP) lysis buffer (Containing a protease inhibitor cocktail). A quantity of 40 mg total protein was mixed with 5 × loading buffer and heated (100°C for 10 min). The protein lysates were electrophoresed in 10% SDS-PAGE gels and transferred onto polyvinylidene fluoride (PVDF) membranes. The antibody against Smad2 (CST, #5339, 1:1000), p-Smad2 (CST, #18338, 1:1000), Smad3 (Abcam, #ab40854, 1:2000), p-Smad3 (Abcam, #ab52903, 1:2000), VEGF (Abclonal, #A12303, 1:500), and β-actin (Sigma, #A5441, 1:10000) were incubated at 4°C overnight. The membrane was incubated with the corresponding second antibody after washing with TBST at room temperature three times. Antibody signals were detected *via* the ChemiDox XRS+ image-forming system.

### RNA Isolation and Quantitative Real-Time PCR

Total RNA was extracted from HUVECs *via* an RNAiso Plus kit (Takara, Japan). The concentrations were measured using a NanoDrop 2000 (Thermo Scientific, USA). gRNA was reverse transcribed into cDNA, according to the manufacturer's protocol using the PrimeScript™RT reagent Kit with a gDNA Eraser (Takara). The quantitative real-time PCR was conducted using a 7,500 Fast Real-Time PCR System (Applied Biosystems, Life Technologies). The following primers were used: VEGF forward sequence, TTGCCTTGCTGCTCTACCTCCA, and reverse sequence: GATGGCAGTAGCTGCGCTGAT; β-actin forward sequence: CACCATTGGCAATGAGCGGTTC and reverse sequence: AGGTCTTTGCGGATGTCCACGT.

### Tube Formation Assay

A total of 100 μL chilled Matrigel was added to a precooled 6-well-plate and solidified at 37°C for 1 h. The HUVEC cells were precipitated by centrifugation and resuspended in HUVEC complete media (1 ml) containing 1% fetal bovine serum. The HUVECs were seeded onto the solidified Matrigel in the 6-well-plates (2 × 10^4^ cells per well). The cells were then supplemented with media from the HUVEC cells treated with TGF-β1 for 24 h or blank and incubated at 37°C in 5% CO_2_ for 4 h. A light microscope was used to capture the image of the tube formation using a magnification of ×40. Image J software was used to count the tubes of the branches and loops.

### Statistical Analysis

The ROC curve was generated *via* the SPSS 21.0 software. The results are presented as means ± SEM, and statistical analysis was performed between the different groups using Student's *t*-test in GraphPad Prism8.0. The values *p* < 0.05 were considered to be significant. ^*^p < 0.05, ^**^p < 0.01, ^***^p < 0.001, and ^****^p < 0.0001.

## Results

### Patient Characteristics

A total of 23 ischemic MMD patients were recruited to the present study, including 9 children (<18 years old) and 14 adults (>18 years old). Among them, 9 patients were women and 14 patients were men. The median age was 29.7 ± 3.6 years and ranged from 7 to 54 years. Transdural collaterals were present in 14 (60.9%) patients. According to the grade of Suzuki, 13 patients in I-III (I, *n* = 0; II, *n* = 5; III, *n* = 8), 10 patients with IV-VI (IV, *n* = 6; V, *n* = 4; VI, *n* = 0); 6 patients presented with transient ischemic attack (TIA) and 17 patients presented with infraction; 13 patients underwent indirect surgery, and 10 patients underwent combined procedures.

### Upregulation of TGFβ1 in the Middle Cerebral Artery of MMD

**Figure 2:**
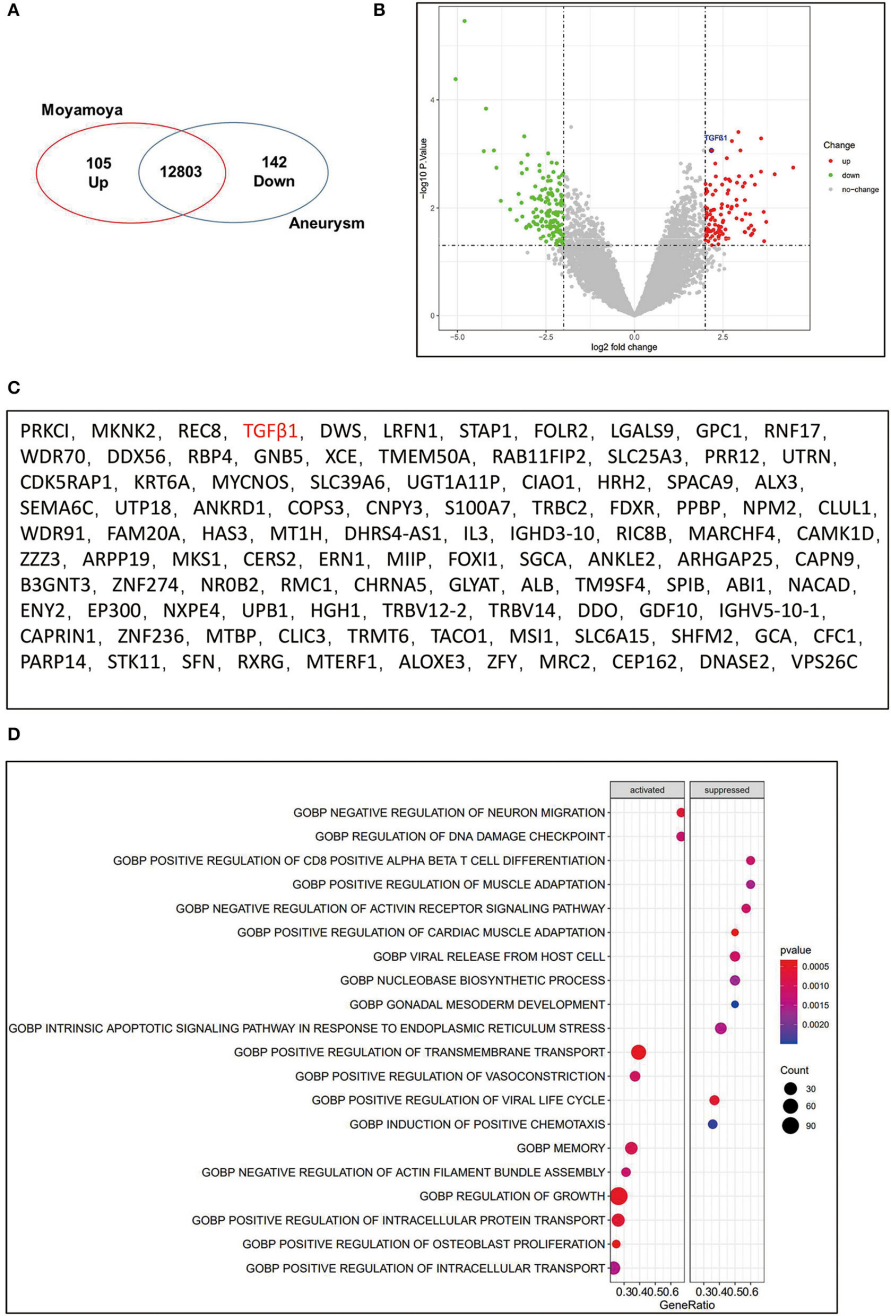
Upregulation of TGFβ1 in the middle cerebral artery(MCA) of MMD. **(A)** Venn diagram showed significantly upregulated genes (*n* = 105) and significantly downregulated genes (*n* = 142). **(B)** Volcano plot to visualize the DEGs in MMD compared to control. The significantly upregulated genes were shown with red dots, and green dots represent significantly downregulated genes. Gray dots represent genes not differentially expressed. **(C)** the upregulated genes were listed. **(D)** GO functional enrichment analysis of sequencing data.

To explore the DEGs in MMD, we analyzed the MCA transcriptomes of patients with MMD compared with those of patients with an aneurysm. We found 105 upregulated and 142 downregulated differentially expressed genes in MMD, which have been visually shown in the volcano plot (Figures 2A–C). According to these DEGs, the GO functional enrichment analysis identified several relatively associated terms, including regulation of cell growth, transmembrane transport, and vasoconstriction (Figure 2D). There is a distinctive characteristic that the development of abnormal vascular networks and collateral formations of the ECA in moyamoya disease have the ability to promote angiogenesis (3, 22). To further explore the mechanisms of the development of angiogenesis, we identified that the angiogenesis-related genes-TGFβ1 were significantly overexpressed in the MCA of MMD (Figure 2B).

### TGFβ1 Associated With Transdural Collaterals in Ischemic MMD

**Figure 3:**
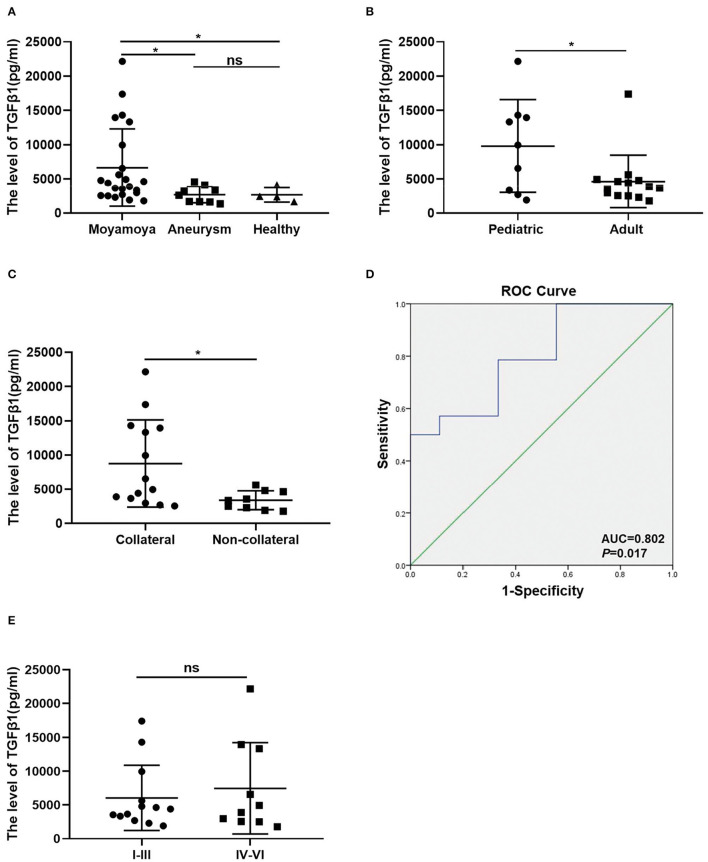
TGFβ1 is associated with transdural collaterals in MMD. **(A)** The levels of TGFβ1 were significantly increased in MMD patients compared to other groups. **(B)** the Expression level of the TGFβ1 in plasma between pediatric group (*n* = 9) and adult group (*n* = 14). **(C)** The different concentration of TGFβ1 between collateral group (*n* = 9) and non-collateral group (*n* = 14). **(D)** The value of the TGFβ1 predicted the collateral formation being analyzed by ROC curves. **(E)** The level of TGFβ1 between Suzuki I-III (*n* = 13) and IV-VI (*n* = 10). ns, non significant (*p* > 0.05), *p < 0.05.

The TGFβ1 was observed to be overexpressed in the MCA of MMD, and the TGFβ1 is secreted as a multifunctional polypeptide growth factor in the plasma (16, 17). ELISA was performed to detect the concentration of TGFβ1 in plasma. Compared to patients with aneurysm and healthy controls, the highest levels of TGFβ1 were detected in the MMD group (6,666 ± 1,181 vs. 2,684 ± 399, 2,651 ± 530, *p* < 0.05) (Figure 3A). In addition, TGFβ1 was determined at a relatively high level in pediatric groups (9,810 ± 2,260 vs. 4,644 ± 1,025; *p* < 0.05) (Figure 3B). Importantly, the concentrations of TGFβ1were significantly increased in the collateral group (8,773 ± 1,708 vs. 3387 ± 460.7; *p* < 0.05) (Figure 3C). The ROC curve demonstrated that the levels of TGFβ1 in plasma predicted the formation of transdural collateral with high sensitivity and specificity. The area under the ROC curve was 0.802 (Figure 3D). But the level of TGFβ1 without a difference between Suzuki I-III and IV-VI (6,047 ± 1,340 vs. 7,470 ± 2,144; *p* = 0.5626) (Figure 3E).

### Association Between VEGF and Transdural Collaterals in Ischemic MMD

**Figure 4:**
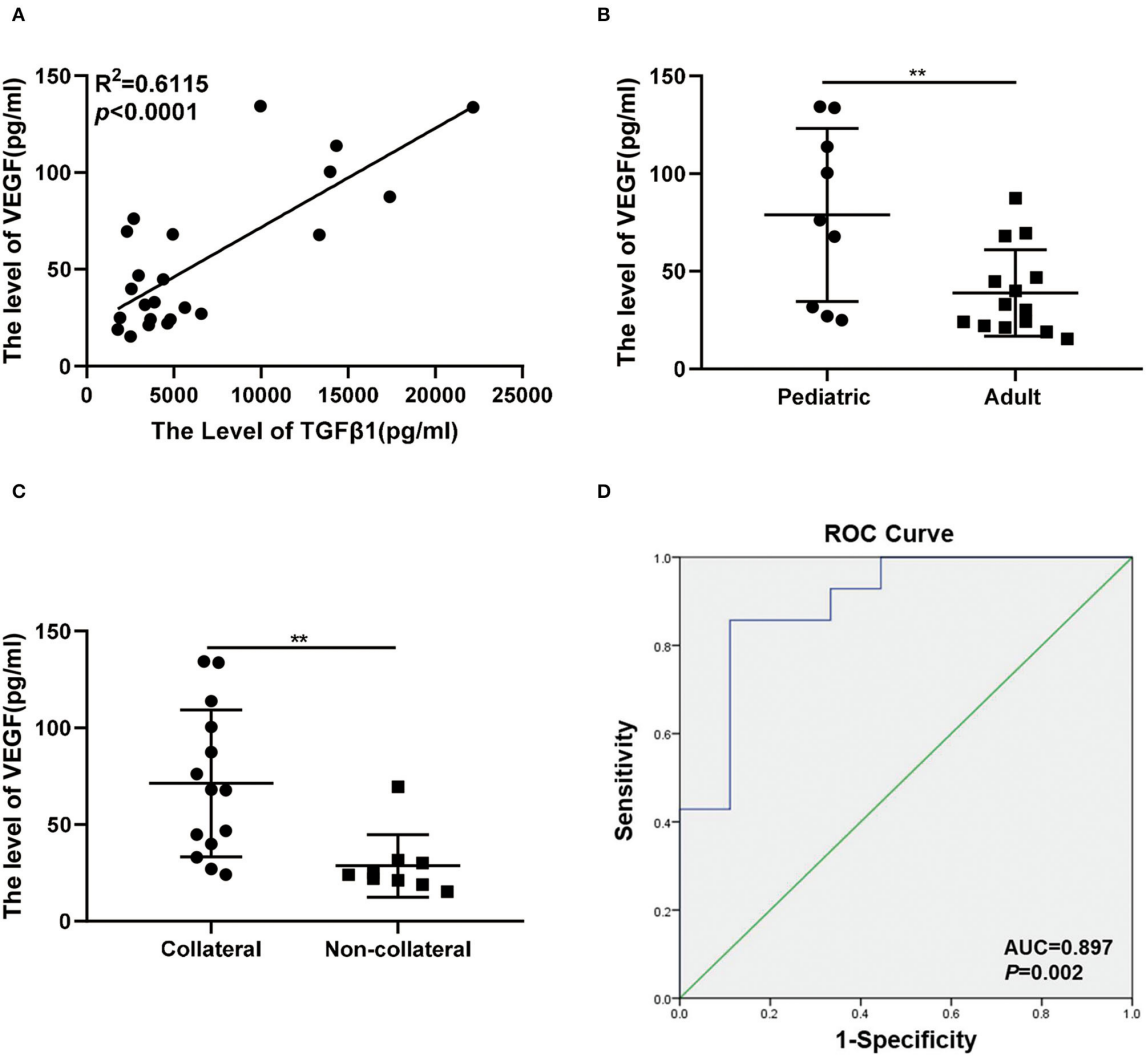
Association between VEGF and transdural collaterals in MMD. **(A)** The levels of TGFβ1 was correlated with VEGF in plasma of MMD under the linear regression analysis. **(B)** There was a higher level of VEGF in pediatric group (*n* = 9) than the adult group (*n* = 14). **(C)** The expression level of VEGF between collateral group (*n* = 9) and non-collateral group (*n* = 14). **(D)** The ROC curves were used to assess the value of the VEGF and predict the collateral formation. **p < 0.01.

In previous studies, VEGF, bFGF, and IL8 are included as direct angiogenic growth factors that can stimulate angiogenesis and promote cellular division in endothelial cells, but the TGFβ1 and PDGF belong to the indirect angiogenic growth factors (15). VEGF is reported to be the target gene of TGFβ1 in tumors and inflammation (23–25). Interestingly, the levels of TGFβ1 had a positive correlation with VEGF in the plasma of MMD using multiple linear regression analysis (Figure 4A). VEGF was detected at higher concentrations within the pediatric groups and corresponded to the trend seen in TGFβ1 (78.88 ± 14.77 vs. 38.97 ± 5.905; *p* < 0.01) (Figure 4B). The levels of VEGF were also significantly upregulated in the collateral group (71.25 ± 10.15 vs. 28.65 ± 5.386; *p* < 0.01) (Figure 4C). The concentration of VEGF was used to predict the development of transdural collateral with high sensitivity and specificity which was demonstrated by the curve of ROC, and the area under the ROC curve was 0.897 (Figure 4D).

### TGFβ1 Upregulated VEGF to Promote the Angiogenesis *via* Activating the TGFβ Signaling Pathway *in vitro*

**Figure 5:**
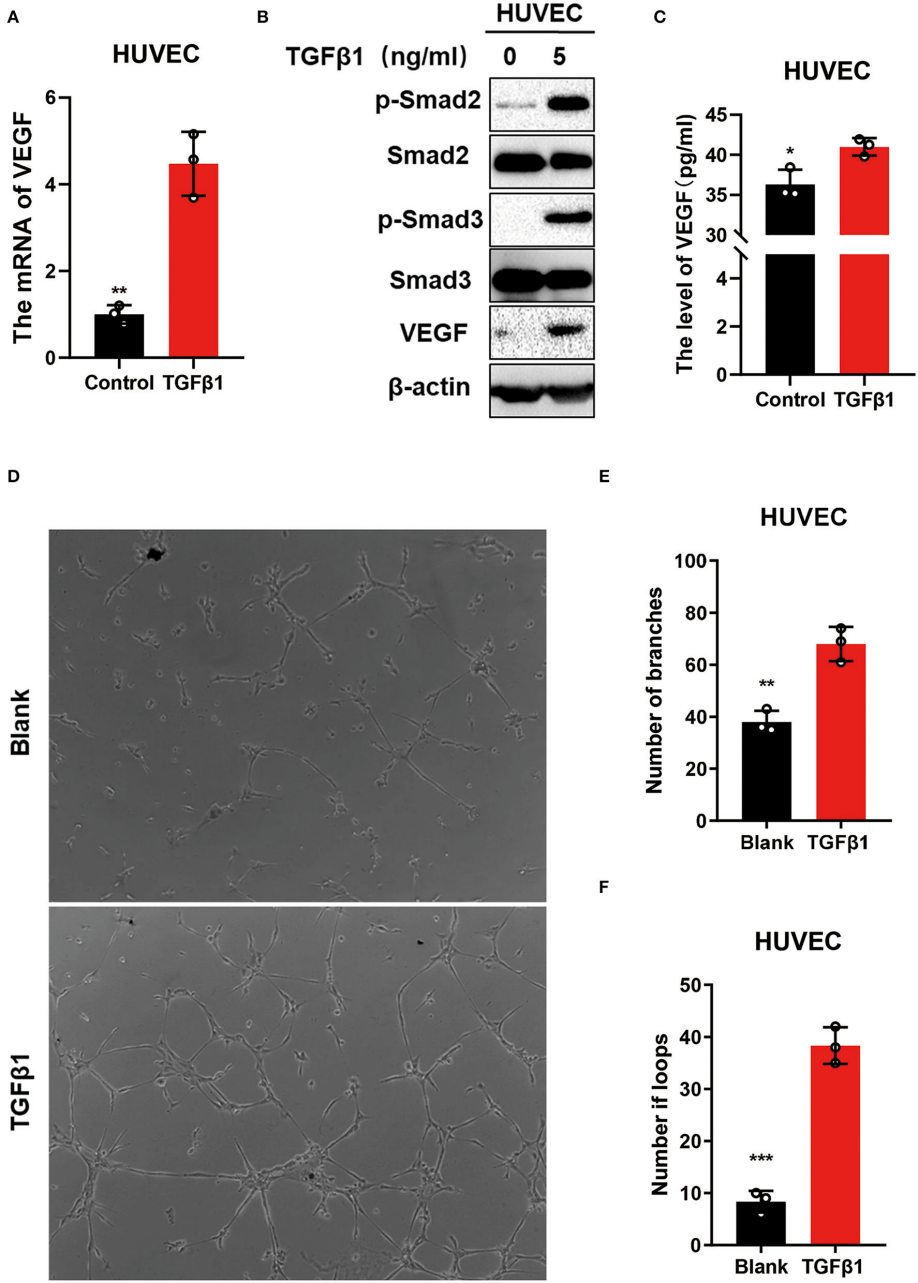
TGFβ1 upregulated VEGF to promote angiogenesis *via* activating the TGFβ signaling pathway *in vitro*. **(A)** The mRNA levels of VEGF in HUVECs after stimulating with TGFβ1(5 ng/ml) for 24 h. **(B)** Western blot was used to detect the molecular of TGFβ pathway. **(C)** ELISA was performed to identify the level of VEGF in medium after the HUVECs were stimulated with TGFβ1(5 ng/ml). **(D–F)** The tube formation assay was conducted to access the ability of angiogenic in which the HUVECs cultured with the medium from the HUVECs treated with TGF-β1. *p < 0.05, **p < 0.01, ***p < 0.001.

The expression of VEGF was analyzed further to identify the mechanisms of TGFβ1 regulation on transdural collaterals. The mRNA and protein levels of VEGF were upregulated in HUVEC cells treated with TGFβ1 for 24 h (Figures 5A,B). Interestingly, the concentration of VEGF in the cell culture media was significantly higher in cultured HUVEC cells treated with TGFβ1 for 24 h compared to control (Figure 5C). Using the tube formation assay, the HUVECs cultured with the media from the HUVEC cells treated with TGF-β1 showed a significantly enhanced ability of angiogenesis (Figures 5D–F).

## Discussion

This study explored the relationship between the TGFβ1 and transdural collateral. The major findings are as follows: (1) the expression of TGFβ1 was upregulated in patients with MMD; (2) the level of TGFβ1 had a positive correlation with VEGF in plasma, which is related to the transdural collateral in patients with MMD; and (3) TGFβ1 upregulated VEGF to promote angiogenesis through the activation of the TGFβ signaling pathway.

The ischemic events in MMD are attributed to reduced blood flow caused by stenosis arteries (26). The collaterals are important to prevent the occurrence of stroke, reduce the incidence of perioperative complications, and are closely related to clinical outcomes (22, 27). Among collateral circulation, the transdural collaterals play the most important role in the collateral blood supply to the ischemic brain cortex (28). In our study, 60.8% (14/23) of the transdural collaterals were established and supplied blood to the cortex.

In the past few decades, the etiology and pathophysiology of MMD are still unclear (1, 22). It is difficult to extract the samples from the patients with MMD, which restricts the research on the molecular mechanisms of collaterals (21, 29). With the development of technology, facilitating the transcriptome analysis (21), this study revealed that the expression of TGFβ1 was upregulated in MCA, as well as within the plasma extracted from MMD patients. Furthermore, the function of TGFβ1 associated with angiogenesis has been studied (23–25). These changes and functions are consistent with the pathological features of spontaneous transdural collaterals that were shown in patients with MMD (7). Moreover, higher levels of TGFβ1 were observed in MMD accompanied by transdural collaterals. These findings demonstrate that the TGFβ1 may be a unique biomarker for the formation of transdural collaterals.

Vascular endothelial growth factor is one of the direct angiogenic growth factors which can stimulate angiogenesis (15). Our present data revealed that the high concentration of VEGF in the patient's plasma indicates better transdural collaterals. In addition, we observed that the level of TGFβ1 positively correlates with the VEGF pathway in MMD. This result suggests that VEGF levels are also related to transdural collaterals and correlate with the TGFβ1 levels. Interestingly, we found that the TGFβ1 upregulated the protein and mRNA levels of VEGF in HUVEC cells *in vitro*. It was also revealed that TGFβ1 can stimulate the HUVEC cells to secret VEGF into the cell culture media. These results suggest that TGFβ1 promotes the expression of VEGF in HUVEC cells. The function of TGFβ1 and the subsequent regulation of VEGF have been reported in other disease states (30, 31). Furthermore, we conducted a western blot analysis and demonstrated that TGFβ1 promotes the phosphorylation of Smad2 and Smad3, which activate the TGFβ pathway in HUVEC cells. Finally, the TGF-β1 was confirmed to enhance the ability of angiogenesis in HUVEC cells; thus, we concluded that TGF-β1 promotes the formation of transdural collaterals *via* the activation of the TGFβ signaling pathway promoting angiogenesis *in vitro*.

In previous studies, the spontaneous transdural collaterals that appear were considered the brain's ability to promote angiogenesis and served as a radiographic biomarker to predict the collaterals' development after revascularization (7, 8, 12). In this research, we detected that the levels of TGFβ1 were associated with the formation of transdural collaterals, indicating that TGFβ1 may become a vital biomarker in predicting collaterals after revascularization. Furthermore, we found that the relatively high concentrations of TGFβ1 were shown in pediatric patients with MMD. Moreover, an excellent postoperative collateral formation was detected in pediatric patients rather than adult with MMD (32, 33). These findings revealed that high levels of TGFβ1 may promote the formation of the collaterals in pediatric MMD patients and further demonstrates that the levels of TGFβ1 are related to collaterals.

Our study has some limitations. First, we identified the TGFβ1 overexpressed in MCA of MMA through bioinformatics analysis, but it was not verified in blood vessel wall tissue due to the specimen being difficult to obtain. Second, the subjects are patients with ischemic moyamoya disease, and there is a lack of the type of headaches and hemorrhage. In addition, the patients and specimens could be affected *via* selection bias because of the single-center study and the limited sample size. Finally, although we speculate that TGFβ1 may be related to the collaterals after the revascularization, we have not reviewed the follow-up data.

## Conclusion

The present study showed that TGFβ1 might play an important role in promoting collateral formation by activating the TGFβ pathway and upregulating the VEGF in ischemic MMD, taking into account the function and mechanism of the TGFβ1, which might be an important target for collateral-enhancing and preventing stroke in ischemic MMD.

## Data Availability Statement

The original contributions presented in the study are included in the article/Supplementary Materials, further inquiries can be directed to the corresponding author.

## Ethics Statement

The Research Ethics Committee approved this study of the Xiangya Hospital. Written informed consent to participate in this study was provided by the participants' legal guardian/next of kin. Written informed consent was obtained from the individual(s), and minor(s)' legal guardian/next of kin, for the publication of any potentially identifiable images or data included in this article.

## Author Contributions

JH designed the study and wrote the manuscript. YC and JW analyzed the data. MT and HLiu collected clinical data. YC and HLi performed *in vitro* experiments. All authors have contributed to the article and approved the final manuscript.

## Funding

This work was funded by the National Natural Science Foundation of China [82160554 (JH)] and Natural Science Foundation of Hunan Province [2020JJ8051 (JH)].

## Conflict of Interest

The authors declare that the research was conducted in the absence of any commercial or financial relationships that could be construed as a potential conflict of interest.

## Publisher's Note

All claims expressed in this article are solely those of the authors and do not necessarily represent those of their affiliated organizations, or those of the publisher, the editors and the reviewers. Any product that may be evaluated in this article, or claim that may be made by its manufacturer, is not guaranteed or endorsed by the publisher.
