# Supplementary material for: TGFβ1 as a Predictive Biomarker for Collateral Formation Within Ischemic Moyamoya Disease
Source: Front Neurol. 2022 Jul 7;13:899470. doi: 10.3389/fneur.2022.899470 (PMC9301205; doi:10.3389/fneur.2022.899470)
Supplement: Supplementary file 1 [file Data_Sheet_1.docx]

**Supplementary Figure**

**Supplementary Figure**

Hemorrhagic moyamoya disease mainly occurs in adults and there is a difference in the level of TGFβ1 between adults and children, so we analyzed the expression of TGFβ1 between ischemic and hemorrhagic in adult moyamoya. We found that without significantly difference in subtype (ischemic &hemorrhage) of adult moyamoya (4644 ± 1025, n=14 versus 4216 ± 661.4, n=4; p= 0.8322).
